# Supplementary material for: Dynamic regulation of CeA gene expression during acute and protracted abstinence from chronic binge drinking of male and female C57BL/6J mice
Source: bioRxiv. 2024 Feb 4:2024.02.02.578650. Preprint. [Version 1] doi: 10.1101/2024.02.02.578650 (PMC10862834; doi:10.1101/2024.02.02.578650)
Supplement: Supplement 1 [file NIHPP2024.02.02.578650v1-supplement-1.pdf]

bioRxiv preprint doi: https://doi.org/10.1101/2024.02.02.578650; this version posted February 4, 2024. The copyright holder for this preprint (which was not certified by peer review) is the author/funder, who has granted bioRxiv a license to display the preprint in perpetuity. It is made available under aCC-BY 4.0 International license.

Supplementary Figures:

bioRxiv preprint doi: https://doi.org/10.1101/2024.02.02.578650; this version posted February 4, 2024. The copyright holder for this preprint (which was not certified by peer review) is the author/funder, who has granted bioRxiv a license to display the preprint in perpetuity. It is made available under aCC-BY 4.0 International license.

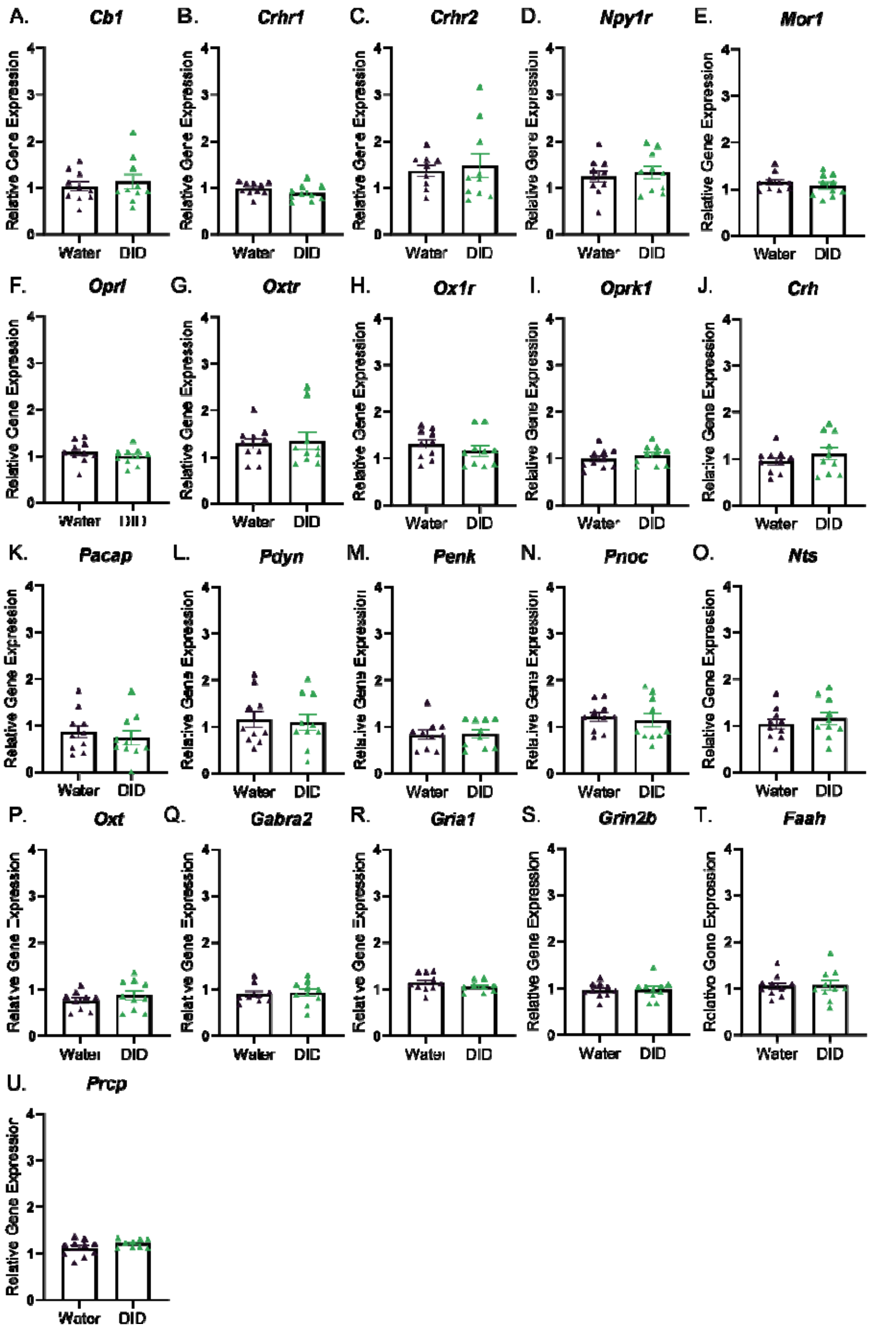

Figure S1: Relative gene expression to housekeeping gene actin B in acute abstinence after DID male mice (A-U) Relative gene expression level of CeA derived genes from male mice from the acute abstinence group (1 day of abstinence after DID) compared to their respective water group. Error bars are depicted as ±SEM.

bioRxiv preprint doi: https://doi.org/10.1101/2024.02.02.578650; this version posted February 4, 2024. The copyright holder for this preprint (which was not certified by peer review) is the author/funder, who has granted bioRxiv a license to display the preprint in perpetuity. It is made available under aCC-BY 4.0 International license.

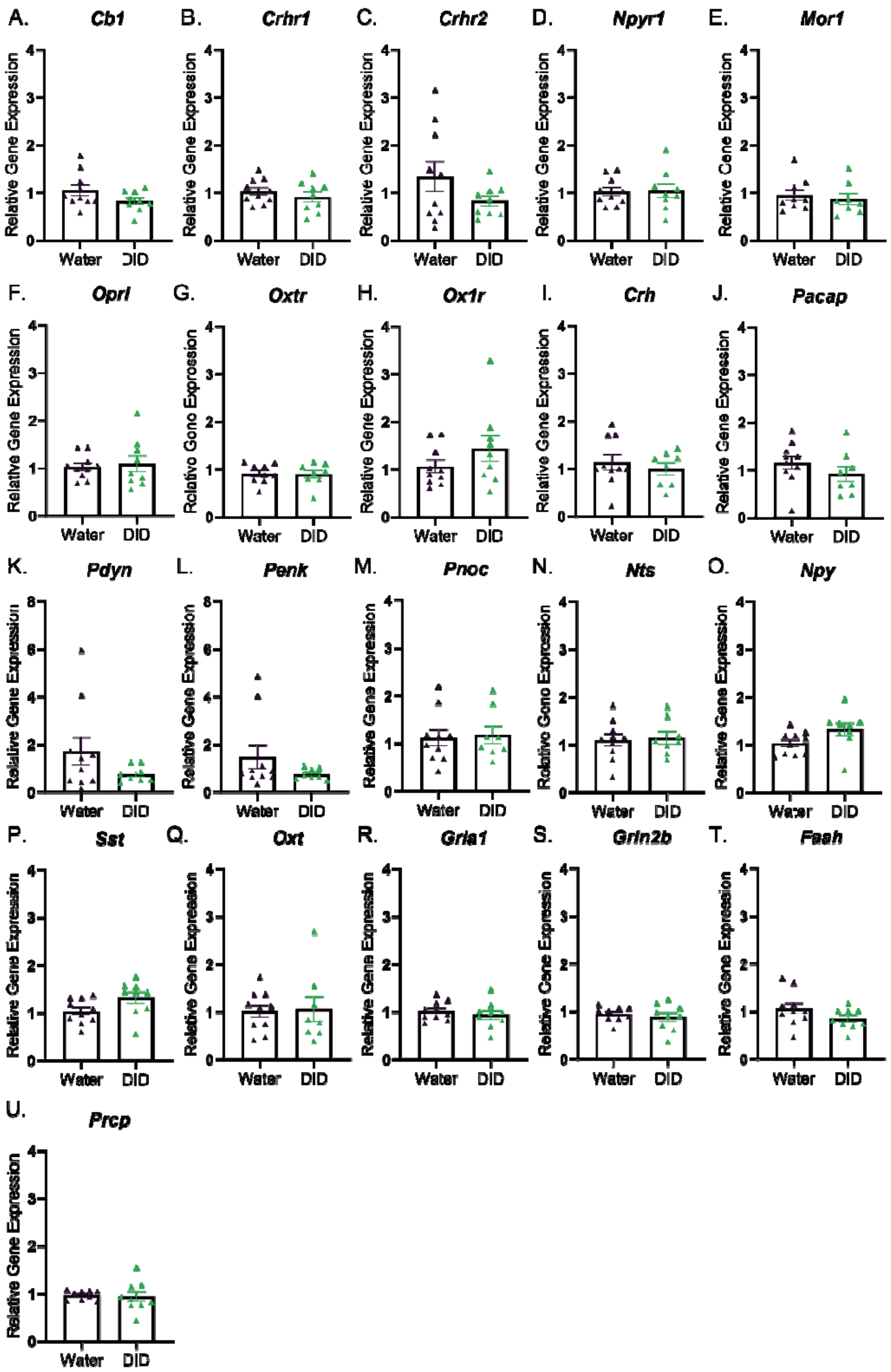

443

Figure S2 Relative gene expression to housekeeping gene actin B in protracted abstinence after DID male mice (A-U) Relative gene expression level of CeA derived genes

bioRxiv preprint doi: https://doi.org/10.1101/2024.02.02.578650; this version posted February 4, 2024. The copyright holder for this preprint (which was not certified by peer review) is the author/funder, who has granted bioRxiv a license to display the preprint in perpetuity. It is made available under aCC-BY 4.0 International license.

from male mice of the protracted abstinence group (7 days of abstinence after DID) compared to their respective water group. Error bars are depicted as ±SEM.

bioRxiv preprint doi: https://doi.org/10.1101/2024.02.02.578650; this version posted February 4, 2024. The copyright holder for this preprint (which was not certified by peer review) is the author/funder, who has granted bioRxiv a license to display the preprint in perpetuity. It is made available under aCC-BY 4.0 International license.

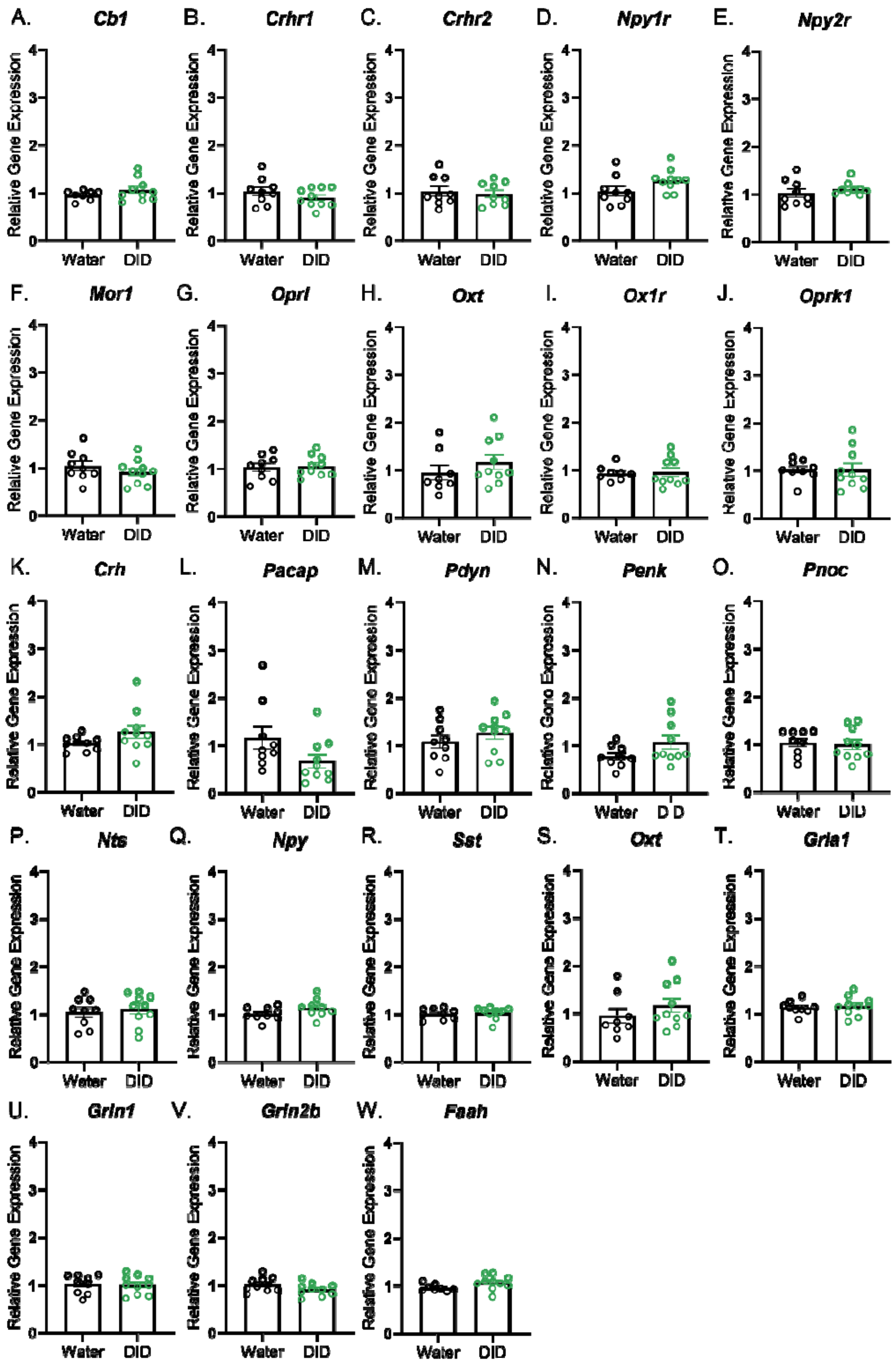

Figure S3: Relative gene expression to housekeeping gene actin B in acute abstinence after DID female mice (A-U) Relative gene expression level of CeA derived genes from the

bioRxiv preprint doi: https://doi.org/10.1101/2024.02.02.578650; this version posted February 4, 2024. The copyright holder for this preprint (which was not certified by peer review) is the author/funder, who has granted bioRxiv a license to display the preprint in perpetuity. It is made available under aCC-BY 4.0 International license.

female acute abstinence group (1 day of abstinence after DID) compared to their respective water group. Error bars are depicted as ±SEM.

bioRxiv preprint doi: https://doi.org/10.1101/2024.02.02.578650; this version posted February 4, 2024. The copyright holder for this preprint (which was not certified by peer review) is the author/funder, who has granted bioRxiv a license to display the preprint in perpetuity. It is made available under aCC-BY 4.0 International license.

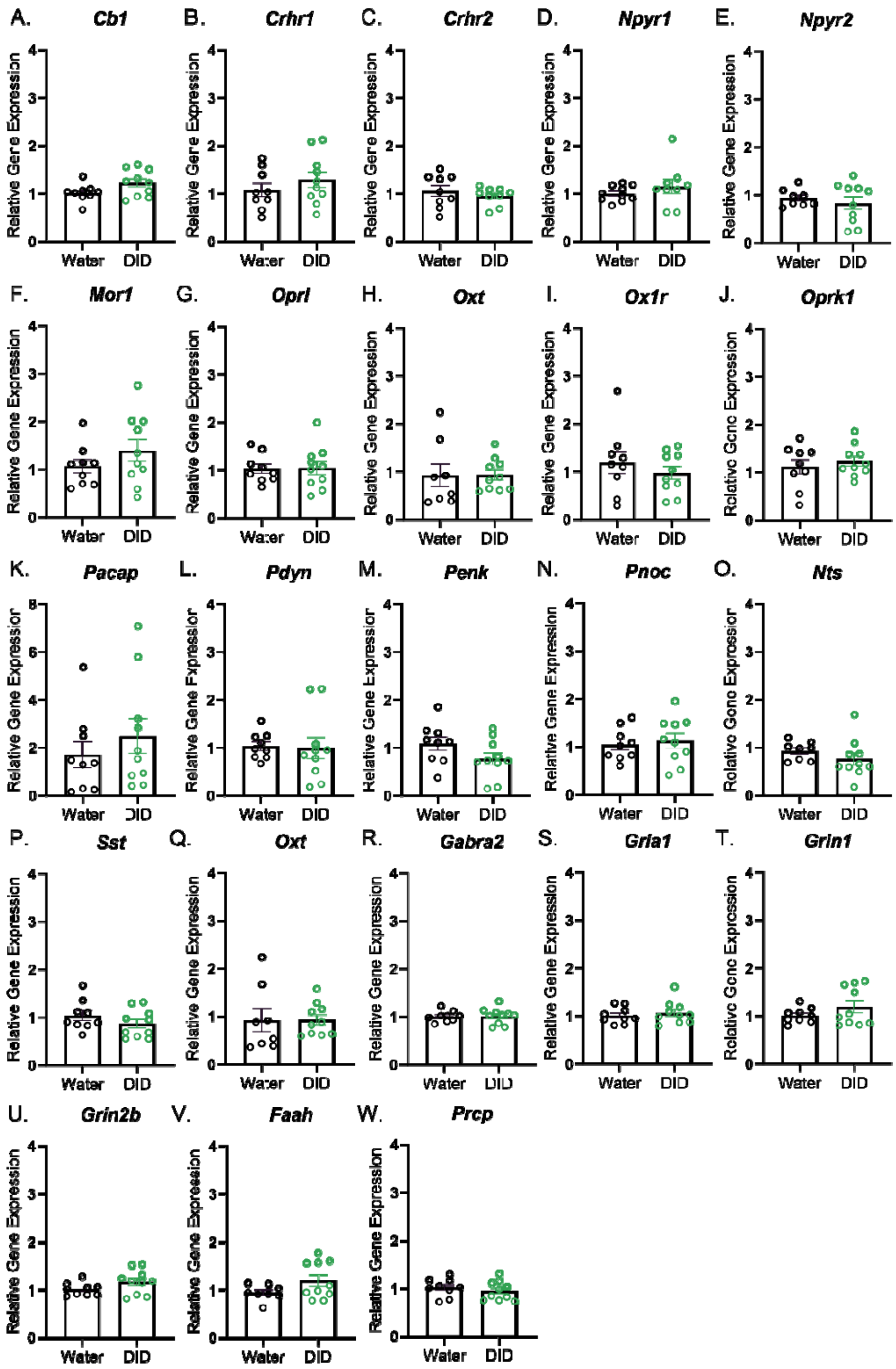

Figure S4: Relative gene expression to housekeeping gene actin B in protracted abstinence after DID female mice (A-U) Relative gene expression level of CeA derived genes

bioRxiv preprint doi: https://doi.org/10.1101/2024.02.02.578650; this version posted February 4, 2024. The copyright holder for this preprint (which was not certified by peer review) is the author/funder, who has granted bioRxiv a license to display the preprint in perpetuity. It is made available under aCC-BY 4.0 International license.

from the female protracted abstinence group (7 days of abstinence after DID) compared to their respective water group. Error bars are depicted as ±SEM.
